# Clinical utility of liquid biopsy in canine oral malignant melanoma using cell-free DNA

**DOI:** 10.3389/fvets.2023.1182093

**Published:** 2023-06-20

**Authors:** Michihito Tagawa, Minori Aoki

**Affiliations:** ^1^Veterinary Medical Center, Obihiro University of Agriculture and Veterinary Medicine, Obihiro, Hokkaido, Japan; ^2^Department of Veterinary Associated Science, Okayama University of Science, Imabari, Japan

**Keywords:** dog, melanoma, liquid biopsy, cell-free DNA, DNA integrity index, LINE-1

## Abstract

**Introduction:**

Cell-free DNA (cfDNA), an extracellular free DNA released into the bloodstream by cells, is a potentially useful noninvasive marker to detect human malignancies and monitor response to treatment. In the present study, we evaluated the utility of circulating cfDNA in canine patients with oral malignant melanoma (OMM) in assessing therapeutic response and clinical outcomes.

**Methods:**

Plasma samples were collected from 12 dogs with OMM and 9 healthy controls. cfDNA concentration was quantified by real-time PCR resulting in short (99bp) and long (218bp) fragments of long interspersed nuclear element-1 (LINE-1), and the DNA integrity index (DII) was then calculated (218/99). A follow-up study was conducted on 6 dogs with OMM, and the plasma cfDNA and DII were quantified throughout disease progression.

**Results:**

Although cfDNA levels obtained from dogs with OMM were not significantly different compared to those obtained from healthy controls, the DII was significantly lower in dogs with OMM than in healthy controls. The DII tended to decrease as the disease stage progressed. Moreover, changes in cfDNA concentration and DII along the clinical course were observed when major changes, such as metastasis or apparent tumor progression, were observed.

**Discussion:**

The results of our study suggest that measurements of serum cfDNA and DII using LINE-1 might be valuable new biomarkers for monitoring OMM progression in dogs. This preliminary study demonstrated the potential clinical utility of monitoring plasma cfDNA in canine patients with OMM.

## 1. Introduction

Canine oral malignant melanoma is an extremely aggressive tumor with a poor prognosis. Early surgical removal is required for long-term survival, with survival time reported to be ≥ 500 days for dogs with stage I tumors (< 2 cm diameter tumor) (1). Although the metastatic rate at diagnosis is 12.9%, local and distant metastases have been reported in ~70–95% of advanced dogs (2, 3). Radiotherapy and chemotherapy have been used as adjunctive treatments for patients with advanced disease (4). Radiation therapy is effective for the local control of tumors, resulting in survival times of 5–11 months (3). However, chemotherapy provides no survival benefit over surgery, and the survival time for oral malignant melanoma (OMM) canine patients with metastasis is ~2–4 months (2, 5). Early diagnosis is paramount to the long-term survival of OMM patients; however, most dogs with OMM are diagnosed at an advanced stage due to the difficulty of routinely examining tumors in the dog's mouth (6). Additionally, there are no tumor biomarkers that can be used to monitor melanoma progression or assess the response to treatment.

Circulating cell-free DNA (cfDNA) is extracellular DNA released into the bloodstream during the turnover of apoptotic and necrotic cells (7). cfDNA is found at low levels in healthy individuals but at high levels in patients with many diseases, including cancer. Changes in plasma cfDNA levels reflect tumor burden and may be used as biomarkers for cancer detection in human and veterinary medicine (8, 9). Analysis of cfDNA has many advantages over conventional tissue biopsies or diagnostic imaging, which require general anesthesia. First, the procedure only requires a blood sample from which plasma or serum is derived for analysis. Second, the use of the quantitative PCR (qPCR) method to detect cfDNA is well-established, rapid, and highly sensitive, requiring only small amounts of starting material. Moreover, qPCR, including the construction of primers for detecting specific cfDNAs, is relatively inexpensive. In conjunction with the measurement of cfDNA, the DNA integrity index (DII), an indicator of the degree of fragmentation of cfDNA, can be used as an additional biomarker for cancer diagnosis and prognosis (10). DII is calculated as the ratio of large to small DNA fragment concentrations of a known gene, wherein the smaller fragments are < 180 bp in size, which corresponds to apoptotic DNA fragmentation size (11). Different origins of cfDNA have been proposed as the cause of changes in the DII. A recent study confirmed the presence of more fragmented cfDNA, and hence a lower DII, in patients with cancer (12).

Our previous reports showed marked changes in serum cfDNA levels in tumor-affected dogs, especially those with lymphomas; however, few studies have been conducted on dogs with solid tumors (9, 13, 14). Long interspersed nuclear element-1 (LINE-1) is an abundant, non-long terminal repeat retrotransposon that is widely distributed in mammalian genomes and is often used to quantify circulating DNA abundance in humans and dogs (9, 15). Thus, the present study aimed to assess the value of quantifying plasma concentration of cfDNA of the LINE-1 and its use as a tumor biomarker for monitoring treatment response in dogs with OMM.

## 2. Methods

### 2.1. Sample collection and processing

Blood samples from 12 dogs with OMM and 9 healthy controls were collected at the Veterinary Medical Center at the Obihiro University of Agriculture and Veterinary Medicine (VMC-OUAVM) between January 2018 and April 2022. All OMM cases were histologically or cytologically confirmed at the VMC-OUAVM, and initial blood sampling was performed prior to any therapeutic intervention. The staging of patients with OMM was performed according to the WHO staging scheme as follows: stage I patients with a tumor diameter < 2 cm, stage II with a tumor diameter of 2 cm to < 4 cm, stage III with a tumor diameter > 4 cm and/or evidence of lymph node metastasis, and stage IV with distant metastasis (16). For some patients, multiple blood samples were obtained at different times to determine whether cfDNA fluctuations correlated with disease progression and treatment response. Evaluation of treatment response and relapse criteria at each visit was performed according to the guidelines described for solid tumors based on physical examination and additional testing at the clinician's discretion (17). The dogs in the control group were evaluated by general physical examination, complete blood count and blood chemistry, and thoracic and abdominal radiographs. No clinically diagnosed malignancies, autoimmune diseases, or inflammation was confirmed at the time of blood collection.

Peripheral blood (2 mL) in ethylenediaminetetraacetic acid (EDTA) was collected from each dog, and the plasma was separated by centrifugation at 2,000 × *g* for 10 min at 4°C, transferred to new tubes, and centrifuged at 16,000 × *g* for 10 min at 4°C to remove cell debris. Plasma was stored at −30°C prior to DNA extraction. All samples were processed within 4 h of blood collection.

### 2.2. Extraction and quantification of cfDNA

Circulating cfDNA was extracted from 500 μL of plasma using the MagMAX Cell-Free DNA Isolation Kit (QIAGEN, Hilden, Germany) according to the manufacturer's instructions. The cfDNA was eluted in 50 μL of elution buffer and stored at −30°C until further analysis.

The plasma cfDNA concentration and DII were determined via qPCR targeting the canine LINE-1 gene (GenBank accession number: AY266086.1); the lengths of the two amplicons were 99 and 218 bp and were obtained using two primer pairs as reported previously (9). The first primer set for LINE-1_(99bp)_ was used to amplify both short (99 bp) and long fragments, whereas the second set of primers for LINE-1_(218bp)_ was used to amplify only the long (218 bp) DNA fragments. The results obtained using the LINE-1_(99bp)_ primers represent the total free plasma DNA, whereas the results obtained using the LINE-1_(218bp)_ primers reflect the amount of DNA mainly released from non-apoptotic cells. Quantification of the 99 bp amplicon represents the total level of cfDNA (ng/mL). The ratio between the absolute concentration of the longer amplicon (218 bp) and the shorter one (99 bp) defined the DII (218/99), which was used to assess cfDNA fragmentation.

The LINE-1 primer sequences were as follows: 5′-AAATGCAATGAAACGCCGGG-3′ (forward for LINE-1_(99bp)_), 5′-TGGGAATGTGAACTGGTGCA-3′ (forward for LINE-1_(218bp)_), and 5′-TCTTTCGTTGGACACCGAGG-3′ (reverse) (9). The specificity and amplified size of the PCR product using the primer set was confirmed by gel electrophoresis. The reaction mixture for qPCR included 500 nM of each primer, 1 μL of cfDNA template, 10 μL of PowerUp SYBR Green Master Mix (ABI; Thermo Fisher Scientific, Inc., Waltham, MA, USA), and 7 μL of distilled water in a final reaction volume of 20 μL. Quantitative PCR was performed with a CFX connect real-time PCR system (Bio-Rad Laboratories, Hercules, CA, USA) using the following protocol: initial incubation at 50°C for 2 min and 95°C for 2 min, followed by 40 cycles consisting of denaturation at 95°C for 3 s and annealing/extension at 60°C for 30 s. A melt curve (60–95°C) was generated at the end of each run to verify the specificity. A standard curve generated by serial dilution (from 1.0 to 10,000 ng/mL) of genomic DNA obtained from peripheral blood leukocytes of a healthy dog was used to determine the absolute equivalent amount of cfDNA in each sample. Each assay was performed in triplicate.

### 2.3. Statistical analysis

The Mann–Whitney *U*-test was used to compare the OMM and control groups. In addition, the survival time of patients with OMM was defined as the time from study entry to the date of death or last follow-up. Survival curves were calculated using the Kaplan–Meier method with median values as the cut-off. Dogs that remained alive at the end of study or those lost to follow-up were censored from the survival analysis. The generalized Wilcoxon test was used to estimate survival. All analyses were performed using JMP 13 software (SAS Institute Inc., Cary, NC, USA). Differences were considered statistically significant if the *p*-value was < 0.05.

## 3. Results

### 3.1. Patient characteristics

In total, 12 patients with OMM and nine healthy controls were enrolled in this study. The 12 OMM patients consisted of 9 male (2 intact) and 3 female (none intact) dogs with a median age of 12 years (range, 6–16 years). The breeds of the OMM patients included miniature Dachshund (five dogs), Toy Poodle, Miniature Schnauzer, Beagle, Shih Tzu, Labrador Retriever, Yorkshire Terrier, and Jack Russell Terrier (1 dog each). The nine healthy patients consisted of one male (one intact) and eight female (five intact) dogs with a median age of 6 years (range, 1–12 years). The breeds of the healthy patients included Beagle (6 dogs) and Labrador Retriever (three dogs). The OMM group was significantly older than the control group (*p* = 0.002). In the 12 OMM patients, 11 dogs were diagnosed histologically and one dog was diagnosed cytologically. Evaluation of pulmonary metastasis was assessed by CT in 7 patients and by three-view thoracic radiography in the remaining patients. In addition, lymph node metastasis was evaluated by cytology in all dogs. According to the WHO staging criteria, two dogs had stage I OMM, six had stage II OMM, two had stage III OMM, and two had stage IV OMM. One dog underwent extended resection including partial mandibulectomy, and two dogs underwent cytoreductive surgery. Radiation therapy was also performed on 10 dogs. NSAIDs were administered to 9 dogs. A dog had advanced metastasis at diagnosis and received no tumor-specific treatment. Patient characteristics are summarized in Table 1.

**Table 1:** Characteristics of oral malignant melanoma (OMM) patients and healthy controls.

**Characteristics**		**OMM (*n* = 12)**	**Control (*n* = 9)**
Sex	Female	3	8
	Male	9	1
Age, years	(Median: range)	12 (6–16)	6 (1–12)
Tumor diagnosis	Histopathology	11	–
	Cytology	1	–
Tumor stage	I	2	–
	II	6	–
	III	2	–
	IV	2	–
Metastasis at diagnosis	Lymph node	2	–
	Lung	2	–
Treatment	Extended resection	1	–
	Cytoreduction	2	–
	Radiation	10	–
	NSAIDs	9	–
	No treatment	1	–

NSAIDs, Non-steroidal anti-inflammatory drugs.

### 3.2. cfDNA and DII

**Figure 1:**
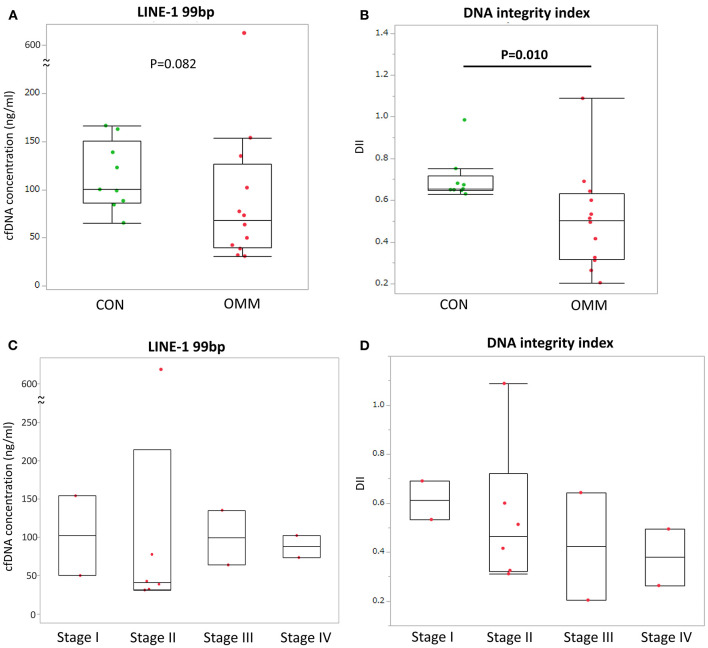
**(A)** Cell-free DNA (cfDNA) concentration of LINE-1 (99 bp) in plasma of dogs with oral malignant melanoma (OMM) and healthy controls (CON). **(B)** cfDNA concentration of LINE-1 (99 bp) in plasma of dogs with OMM grouped by tumor stage. **(C)** DNA integrity index (DII) in plasma of dogs with OMM and healthy controls. **(D)** DII in plasma of dogs with OMM grouped by tumor stage. All blood samplings were performed before any tumor-specific treatment. Each box plot indicates the 25th and 75th percentiles. The horizontal line inside the box indicates the median value, and the whiskers indicate the extreme measured values. The *p*-values and R-squared values are shown.

The median cfDNA concentration of LINE-1_(99bp)_ in the OMM and control dogs were 68.5 ng/mL (mean range, 31.0–623.9 ng/mL) and 154.9 ng/mL (range, 81.0–242.0 ng/mL), respectively (Figure 1A). No significant difference in cfDNA concentration was observed between OMM and control dogs (*p* = 0.082). In contrast, the median DIIs in patients with OMM and control dogs were 0.50 (range, 0.20–1.09), and 0.68 (range, 0.63–0.98), respectively (Figure 1B). The DII was significantly lower in dogs with OMM than in healthy controls (*p* = 0.010). In terms of OMM cfDNA levels and clinical stage, no clear correlation was found between the stage and cfDNA concentration. In contrast, the DII decrease with tumor stage progression in the OMM group (Figures 1C, D).

### 3.3. cfDNA and survival

**Figure 2:**
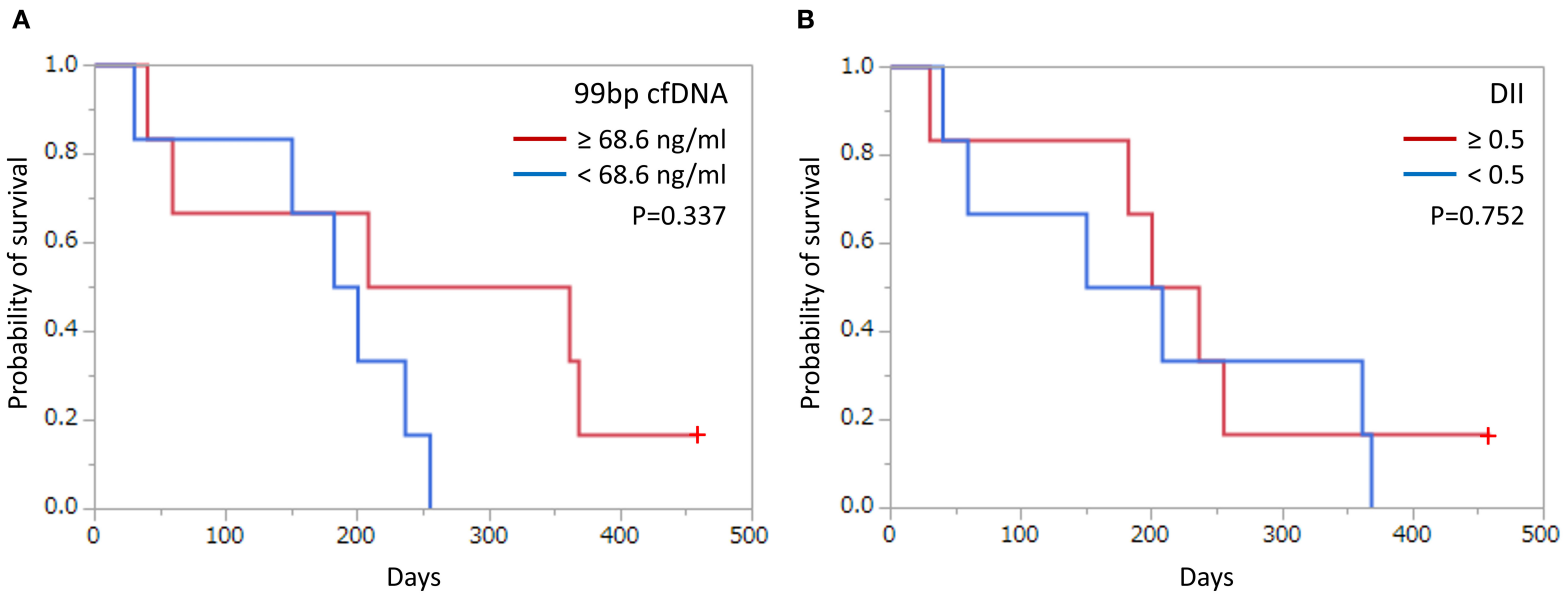
Kaplan–Meier curves of survival time in dogs with oral malignant melanoma according to cut-off values based on **(A)** cell-free DNA (cfDNA) concentrations and **(B)** DNA integrity index (DII). Cut-off and *p*-values are shown. +: Censored case.

All deaths were related to tumor progression in every case except for one case which is still alive at the end of data analysis. Patients with OMM were divided into two groups according to their plasma concentrations of cfDNA and DII at the first sampling. These thresholds were selected based on median values. Neither cfDNA concentration nor DII showed a significant difference in the survival time between the groups (Figure 2).

### 3.4. Changes in cfDNA during treatment

**Figure 3:**
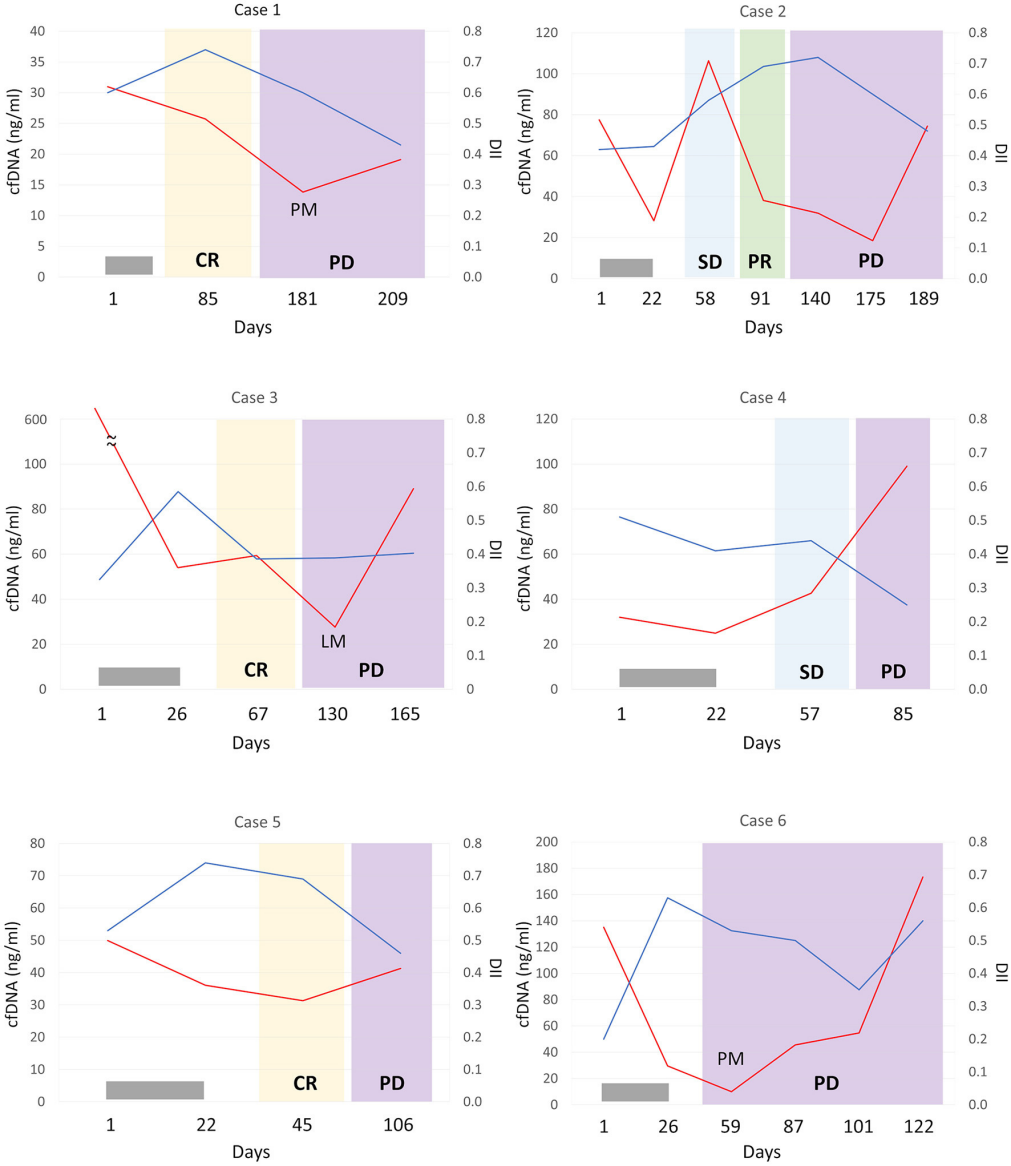
Monitoring of cell-free DNA (cfDNA) concentrations (red lines) and DNA integrity index (DII; blue lines) in plasma from dogs with oral malignant melanoma according to treatment response. In each case, the patient was administered orthovoltage radiation treatment (gray bar); all cases were treated with non-steroidal anti-inflammatory drugs except for case 6 after radiation therapy. CR, complete response; PR, partial response; SD, stable disease; PD, progressive disease; PM, pulmonary metastasis; LM, lymph node metastasis.

To determine the predictive value of cfDNA in canine OMM patients, changes in the concentration of cfDNA and DII during the treatment course were analyzed in six OMM patients at several time points. All 6 patients were treated with orthovoltage radiotherapy, and the protocol involved 8 Gy once a week for 4 weeks for a total dose of 32 Gy. Radiotherapy resulted in decreased cfDNA levels in all dogs and increased DII levels in four of the dogs (Figure 3). Five of the six dogs were administered with regular-dose non-steroidal anti-inflammatory drugs (case number 1, 3, 4, and 5 were on firocoxib and case number 2 was on piroxicam) after radiotherapy for palliative use until the case died. The clinical course and cfDNA variation in the individual cases were as follows.

Case 1 showed complete remission of the tumor after radiotherapy. At that time, a mild decrease in cfDNA and an increase in DII were observed. Lung metastasis was observed on day 181, but no increase in cfDNA concentration was observed. Progression of lung metastases was observed, and DII progressively decreased.

Case 2 showed a decrease in cfDNA concentration after radiotherapy. On day 58 of stable disease (SD), there was a transient increase in cfDNA levels, but on day 91 of PR, cfDNA levels decreased. Regrowth of the primary tumor was observed on day 140, without an increase in cfDNA levels at that time. Subsequently, the primary tumor continued to grow, and an increased cfDNA level was observed on day 189. DII increased after radiation treatment and decreased after day 175 when the primary lesion was clearly re-enlarged.

Case 3 had a marked elevation in cfDNA concentration at diagnosis, but its cfDNA level decreased after radiotherapy. No recurrence of the primary lesion was observed, but lymph node metastasis was confirmed on day 130. However, there was a further decrease in cfDNA levels and no change in DII. cfDNA levels increased on day 165 when the lymph node metastasis worsened.

Case 4 had a primary lesion that was refractory to radiotherapy but became SD after the completion of radiation. On day 85, the primary tumor size increased, and the cfDNA level was also elevated. The DII did not increase even after radiotherapy and markedly decreased with tumor progression.

Case 5 showed complete remission with radiotherapy with decreased cfDNA concentration and increased DII observed. However, the primary lesion recurred on day 106, the cfDNA level was increased, and the DII decreased.

Case 6 achieved complete remission of the primary lesion with radiotherapy. Early pulmonary metastasis was confirmed on day 59, despite the complete response of the primary lesion to treatment, with a dramatic decline in cfDNA level. Subsequently, as lung metastasis worsened, cfDNA level increased, while DII leveled off. In addition, re-enlargement of the primary tumor was observed on day 122.

## 4. Discussion

In the present study, we evaluated the potential of cfDNA in plasma from dogs with OMM as a tumor biomarker for monitoring purposes. The concentration of cfDNA was measured by LINE-1 qPCR at different disease stages and in healthy controls. The use of the LINE-1 gene, a retrotransposon that constitutes 17% of the human genome overcomes the limitation of targeting genes with limited copy numbers and enables the highly sensitive amplification of gDNA (18). However, quantification of the whole cfDNA concentrations, including the LINE-1, may result in non-specific increases due to factors such as tissue injury and/or inflammation (9). Various techniques, including UV spectrophotometry, fluorescence-based nucleic acid quantification, and qPCR, have been reported to quantify and evaluate cfDNA. In addition, it may be possible to capture changes in cfDNA fragmentation patterns by using automated microfluidic capillary electrophoresis assays (19). Although the application of liquid biopsy using cfDNA based on next-generation sequencing for the detection of cancers, including malignant melanoma, in dogs has recently been reported, therapeutic monitoring of malignant melanoma has not been performed (20). Measurement of cfDNA by qPCR is a rapid, easy, and cost-effective quantitative method that is suitable for repeated measurements and can also capture changes in cfDNA fragmentation. Therefore, we determined cfDNA using qPCR with primers targeting LINE-1 to detect and monitor the tumor. To the best of our knowledge, this is the first study to use quantification of cfDNA and the DII assay based on the LINE1 gene as a liquid biopsy for OMM in dogs.

In this study, we found that plasma cfDNA concentration was not elevated in dogs with OMM compared with that in healthy controls and was not associated with survival time. Rapidly systemic spread tumors, such as hematologic malignancies, have shown marked changes in serum cfDNA levels (9, 14). In contrast, in a study using PTPN11 as a reference for quantification, the cfDNA level in dogs with malignant melanoma was lower than that in healthy dogs and was not associated with tumor stage progression (20). In a study on cats with feline diffuse iris melanomas, cfDNA was not elevated in tumor-bearing cats, suggesting that cfDNA released from localized solid tumors may be too low for detectable effects on total cfDNA amounts in the bloodstream of the patients (21). DNA integrity index is a measure of the degree of fragmentation of cfDNA, calculated as the ratio between long and short cfDNA fragments. In our study, a significant decrease in the DII was observed in patients with OMM. Circulating DNA fragments resulting from apoptosis are 160–180 bp in length and mainly arise from normal cell turnover. In contrast, DNA fragments released from necrosis can reach up to several kilobases and are mainly released into the bloodstream by tumor cells. Therefore, analysis of cfDNA fragmentation could potentially identify the source of circulating DNA in patients with cancer, and DII has been recently proposed as a promising specific oncological biomarker in several malignancies (22, 23). Recent studies have reported that circulating tumor DNA from patients with cancer is shorter and more fragmented than circulating DNA from healthy individuals (12, 24). In human patients with breast cancer, a low DII was more frequent in the plasma of patients with metastatic-stage cancer than in those with earlier-stage cancer (25). In addition, patients with hepatocellular carcinoma have a significantly lower plasma DII than those with liver cirrhosis (26). However, research on circulating cfDNA fragmentation and/or DII in dogs with cancer is extremely limited. The DII of dogs with mammary tumors was lower than that of dogs with other diseases or were healthy. Moreover, dogs with malignant mammary tumors have a significantly lower DII than those with benign lesions or healthy individuals (27). In our previous study, decreased DII was observed in dogs with various tumors and distant metastases (9). Although no association was found between DII and tumor stage or survival time, the measurement of DII in the plasma shows potential as a biomarker in dogs with OMM. Further studies with larger sample sizes and standardized treatment modalities are necessary to avoid type II error and to validate our findings.

Notably, although plasma cfDNA concentrations at diagnosis in dogs with OMM were not elevated compared to those in healthy controls, they were correlated, to some extent, with the clinical course in all monitored cases. In addition, DII often exhibited an opposite trend to the changes in cfDNA concentration. However, in cases 1, 3, and 6, cfDNA concentrations did not increase when new metastases were detected, and cfDNA levels increased as the metastatic lesion progressed. Further studies are required to better evaluate the usefulness of cfDNA as a biomarker of canine OMM.

In the present study, the control and patient groups were not age-matched; the healthy control group was significantly younger than the OMM patient group. The age difference is a limitation of this study, potentially representing an objective bias of the study results. In humans, data on age-associated alterations in cfDNA levels are equivocal. Jylhävä et al. (28) observed that healthy women over 90 years of age had elevated plasma cfDNA concentrations compared to those in younger women between 22 and 37 years of age (28). The report showed that age-related changes in cfDNA affect not only the quantity but also the quality of the cfDNA (28). Other reports have shown increased plasma cfDNA values in young and older women but decreased values in middle-aged women (29). In addition, the origin and nature of cfDNA remain unclear. In veterinary medicine, changes in cfDNA levels with aging in dogs have not been investigated. The results of our study may be affected by age; however, age in the control group did not correlate with cfDNA levels or DII (data not shown). Further investigations are needed to determine the effect of age on cfDNA quantity and quality in dogs. In addition, stability of the test is a potential concern for quantification of cfDNA by qPCR. In this study, experiments were conducted in triplicate, but further examination is required regarding the reproducibility of the test results.

Circulating tumor DNA (ctDNA) measurement has become mainstream in human medicine because ctDNA contains the same genetic mutations present in the original tumor, allowing assessment of tumor characteristics and providing enhanced surveillance during cancer treatment beyond the resolution of conventional imaging (30). For example, approximately half of the human patients with melanoma harbor BRAF^V600^ mutations, and highly sensitive detection of minimal residual disease using mutated ctDNA enables clinical course monitoring and prognostic evaluation of these cases (31). In contrast, although canine malignant melanoma is considered a comparative model for human melanoma, little is known about its genetic etiology (32). Targeted sequencing studies have shown that known driver mutations in human melanoma are rare in canine melanoma, and studies of ctDNAs in canine melanoma are extremely limited (32). A recent study revealed that tumor-specific copy number alterations were detectable in the plasma of only 25% of dogs with OMM (20). In recent years, it has become possible to detect ctDNAs in the field of veterinary medicine (20, 33, 34); therefore, it is necessary to establish a method for monitoring ctDNAs, which are highly characteristic of tumors, even malignant melanoma.

## 5. Conclusions

The present study indicates that the measurement of serum cfDNA and DII using LINE-1 might be valuable new biomarkers for monitoring OMM progression in dogs. To the best of our knowledge, this is the first report on the therapeutic monitoring of OMM using liquid biopsy. Monitoring of cfDNA and DII may contribute to the optimization of advanced individualized veterinary medicine for OMM. Future studies on large numbers of canine OMM patients with adequate follow-up times are needed to further validate the clinical utility of serum cfDNA and DII.

## Data availability statement

The original contributions presented in the study are included in the article/supplementary material, further inquiries can be directed to the corresponding author.

## Ethics statement

The animal study was reviewed and approved by the Institutional Animal Care and Use Committee of the Obihiro University of Agriculture and Veterinary Medicine (permission number: 18-2, 21-124). Written informed consent was obtained from the owners for the participation of their animals in this study.

## Author contributions

MT conducted the study, collected samples, reviewed, and revised the manuscript accordingly. MT and MA analyzed the samples, performed data analysis, and drafted the manuscript. All authors have read and approved the final manuscript.
